# Emotional Intelligence and Executive Functions in the Prediction of Prosocial Behavior in High School Students. An InterDisciplinary Approach between Neuroscience and Education

**DOI:** 10.3390/children8090759

**Published:** 2021-08-31

**Authors:** Luis Espino-Díaz, Gemma Fernández-Caminero, Carmen-María Hernández-Lloret, Hugo González-González, José-Luis Álvarez-Castillo

**Affiliations:** Faculty of Education Sciences, University of Cordoba, 14071 Córdoba, Spain; gemma.fernandez@uco.es (G.F.-C.); m32hellc@uco.es (C.-M.H.-L.); hugo.gonzalez@uco.es (H.G.-G.); jlalvarez@uco.es (J.-L.Á.-C.)

**Keywords:** prosocial behavior, emotional intelligence, executive functions, predictive study, neurosciences

## Abstract

One of the most analyzed variables in educational research is prosocial behavior, given its relevance and its application in favor of a culture of peace, the construction of solid democratic societies and the development of social justice. For this reason, it is necessary to extend the knowledge of predictors of prosocial behavior so that they can be transferred to teaching practice. The research reported here was carried out using a quantitative methodology through a survey, based on data collection instruments, whose data were treated statistically by means of descriptive, correlational and predictive analyses. The results obtained suggest that emotional intelligence has a strong predictive capacity for prosocial behavior while executive functions show a mild-moderate predictive behavior. In the conclusions and discussion, we provide a series of arguments on some of the results obtained in contrast with previous literature, as well as incorporating limitations of the study and prospects for future research.

## 1. Introduction

One of the issues of greatest concern in the field of educational research is prosocial behavior (PSB), partly as a consequence of the need to develop from teaching practice the internalization of values that enable a culture of peace and the construction of solid democratic societies. In this line, international institutions such as UNESCO emphasize the importance of education contributing to the development and promotion of learning values, attitudes and behaviors [1] necessary for coexistence and mutual respect. Underlying all this is the impetus of the 2030 Agenda for Sustainable Development in terms of the consolidation of democratic societies [2].

Therefore, the justification of this research is based on investigating possible new predictors of PSB and thus extending the number of variables that can predict this type of behavior. On the other hand, given the enormous importance of neurosciences for its applications in different areas of life, two neuropsychological variables that have been investigated for their possible predictive ability on the educational variable of PSB, according to previous literature, are emotional intelligence (EI) and executive function of the brain (EF).

Numerous investigations have highlighted the importance of EF due to its high impact on several variables connected to the educational field such as academic performance [3], values education [4] or language learning [5], among others. This is also the case with EI, which has been shown to be a construct involved in a multitude of processes [6].

Within this framework, the present research analyzed the PSB of a sample composed of secondary education students. It was a descriptive, correlational and predictive study that aimed to identify some factors that present predictive capacity in adolescents.

The applied scope of this study was focused on the improvement of PSB, focusing on the predictors detected by the research, for their possible inclusion in teaching practice through their presence in teaching programs.

The following is a review of the current status of this field of research. First, the link between PSB and the sociodemographic variables of age and sex is reviewed. Next, previous research that has connected PSB and EF is highlighted. Finally, a review of the literature on the relationship between PSB and EI is conducted.

Prosocial behavior encompasses actions that benefit another person, such as helping, sharing, showing consideration and concern and making amends for past mistakes [7,8]. The linkage between PSB and gender and age is discussed below.

Numerous studies report that, in general, girls score higher on PSB, and, with respect to age, the literature reviewed reports that the older the girl, the higher the score on PSB. This is the case of a study developed in Spain with a sample of undergraduate students of social work in which PSB was measured. The results show higher levels of prosociality in female students and in older students corresponding to the later courses of the degree [9]. Along the same lines, a study on a sample of adolescents reported a significantly higher score in females than in males in the PSB assessment; however, in the case of the sociodemographic variable of age, participants aged 12–15 years scored higher on PSB than participants in the age range between 16 and 18 years [10].

One of the factors associated with EFs in the research setting is PSB. The construct of EF can be defined as a specific set of attentional regulation skills involved in the conscious resolution of goal-directed problems. These skills include cognitive flexibility, working memory and inhibitory control [9]. This conceptualization is considered by other authors as a psychological construct, which refers to the cognitive processes that are required for the conscious and top-down control of action, thought and emotion and that are associated with neural systems involving the prefrontal cortex [11].

On the other hand, it is a multidimensional construct that has been analyzed in its structure by several studies. In this sense, there is consensus in affirming that EF consists of three basic elements such as behavioral regulation, the metacognitive component and emotion regulation [12,13,14,15]. The behavioral dimension, according to these authors, is composed of factors such as inhibition and behavioral monitoring; the metacognitive aspect is formed by the factors of working memory, initiative, planning, organization of materials and task monitoring; and emotion regulation consists of the elements of emotional control and flexibility.

Regarding the importance of EFs [16], the theoretical review suggests that the ability to think, plan and control our thoughts and actions in almost all situations of daily life translates into better decisions and ultimately better relationships and job performance [17,18,19].

A study conducted on the early childhood education stage [20] revealed that prosocial behaviors promote EF. In the same vein, another study that aimed to investigate the role of EF in explaining prosocial behavior through a predictive model showed that EF task performance was directly related to prosocial behavior [4]. 

Recently, research has been published [21] in which the mediating role of EFs between mother–child interaction frequency and school-aged children’s prosocial behavior was considered. Specifically, this study sought to examine through structural analysis the mediation effect of EF and language skills difficulties on the relationship between mother–child interactions and prosocial behavior of secondary–primary school children. The results suggest the benefits of an intervention to promote the development of prosocial behavior among early school-age children, focusing on the improvement of children’s EF and language skills for the development of prosocial behavior. The authors highlighted the need to develop a national intervention program for the EF difficulties of early-school-age children to promote prosocial behavior. Another study analyzed the predictive ability of EF on physical aggression in children aged 9–15 years [22]. Results obtained through linear regression report that metacognitive skills intrinsic to EF and behavioral regulation served as significant predictors of prosocial skills.

Along these lines, the links between EF, Theory of Mind, physical and relational aggression and prosocial behavior have been investigated [23]. The study concluded that EF and Theory of Mind predicted physical aggression but not relational aggression. The authors concluded that poor inhibition as a factor of EFs and delayed gratification is uniquely associated with greater physical aggression.

Similarly, in a sample composed of at-risk adolescents, research was conducted to assess the nature of the connections between perceived patterns of caregiving experienced in childhood, EFs and antisocial behaviors [24]. Structural equation modeling was employed to examine whether EFs mediated the relationship between children’s perceived maternal caregiving patterns and the later development of antisocial behaviors. The results report that maternal care predicted unique variance in both EFs and antisocial behavior. Accordingly, the authors suggested a relationship between the experience of caregiving in childhood and EFs and delinquency in adolescence and highlighted the importance of early parenting interventions to aid the development of EFs to enhance long-term prosocial behavior.

Regarding EI and its connection with PSB, first of all, it is important to highlight that the literature review on the concept of EI reports that the definitions of this construct tend to be varied and, in turn, tend to complement rather than contradict each other. In general, it can be affirmed that there is a consensus in including in the construct four distinct areas: perception, regulation, understanding and use of emotions [25,26,27]. EI and PSB are two constructs with a high level of application in the educational field, although interest in them is relatively recent [22,28,29]. These authors argue that the reason for this late interest is due to the fact that there is currently a tendency to attend to the integral development of students as opposed to a traditional teaching–learning process that tries to reject any element with emotional links. 

On the other hand, prosocial behavior encompasses actions that benefit another person, such as helping, sharing, showing consideration and concern and making amends for past mistakes [7], and brings benefits both at the level of micro contexts (such as family and school) and at the level of macro contexts (more complex social structures). For this reason, educational innovation is trying to increase its presence in educational contexts under various names, from specific activities to larger projects [28]. In fact, these authors developed a quantitative study that responded to a correlational and predictive design focused on the analysis of the relationship between these two constructs. The results obtained report medium-high levels of EI and PSB in a sample of primary school children. In addition, correlational analyses showed the existence of a positive relationship between EI and PSB, and, from a predictive perspective, interpersonal EI is reported as a predictor of PSB.

In terms of predictive studies, prosocial behavior has been analyzed in socioeconomic games, together with spontaneous expressions of emotion [22]. Analysis using structural equations revealed no significant relationship between general EI and prosocial behavior. However, the study of specific emotional factors reported that more prosocial individuals score higher on emotion recognition with a tendency to show a greater ability to express more spontaneous emotions. 

Another approach under analysis has focused on the mediating role of PSB between EI and positive and negative affect in a sample of adolescents [6]. The results report that EI was positively related to positive affect and negatively related to negative affect. On the other hand, the influence of EI on positive affect was fully mediated by prosocial behavior. 

Along the same lines, a study was developed in a sample of Canadian students between 12 and 16 years of age [30]. The object of analysis aimed to extend a line of research by focusing on the enhancing effect of EI and its moderating effects on the relationship between parental parenting and adolescent prosocial behavior. Results indicate that adolescents with high EI who were exposed to positive parenting experiences in early adolescence reported higher levels of prosocial behavior two years later compared to respondents with low or medium EI scores.

To sum up, the object of study of the present research focused on whether neuropsychological variables (EF and EI) predict PSB in the 12- to 16-year-old stage.

## 2. The Present Study

After reviewing the relationships between the different variables that have been taken into account in the theoretical foundation and after an exhaustive search of the previous literature, we highlight the research by Riccio et al. [31] focused on predictive models of EF on PSB and by Kaltwasser et al. [22] that established EI as a predictor of PSB. Therefore, it can be affirmed that no previous studies were found in which EF and EI had been analyzed in the same research. What is needed now is to confirm the predictive ability of EF and EI on PSB in the same investigation. The hypothesis was that it would be achieved by means of positive-valence regression coefficients. On the other hand, this study also aimed to confirm the relationships of sociodemographic and neuropsychological variables with PSB, based on the hypothesis that they would correlate positively with PSB.

Given the importance of PSB for society in general and for education in particular, there is a need to extend predictive knowledge on this variable due to its impact on the implementation of a culture of peace and for the coexistence and development of a democratic society. To address this interest, we chose to investigate the impact of neuropsychological variables such as EF and EI on PSB.

## 3. Methodology

### 3.1. Design

This was a cross-sectional study based on three questionnaires, the aim of which was to explain the connections between the research variables. Consequently, a descriptive, correlational and predictive design was carried out. In the first instance, a descriptive study of the data collected on the variables of sex, age, EI and EF was carried out in order to, in the second phase, locate the variables that were correlated with PSB. Subsequently, the aforementioned variables were subjected to regression analysis as independent variables, and PSB was introduced as a criterion variable.

The collection of information in this study modality was a survey study, specifically, by means of three standardized instruments on PSB, EI and EF, the participants being secondary education students. Following the description of the aforementioned constructs, statistical correlation and regression analyses were carried out.

### 3.2. Participants 

The informant group consisted of 161 male and female students from a secondary school in the province of Córdoba (Spain) (*M_age_* = 14.1, *SD* = 1.18; 46.6% female and 53.4% male). The type of sampling adopted was non-probabilistic purposive, and the data were collected during the month of May 2021. The frequency of 12-year-old students was 11.8%; 13-year-olds, 18.0%; 14-year-olds, 28.6%; 15-year-olds, 30.4%; and 16-year-olds, 10.6%.

For the selection of the groups, the criteria used were that there should be a representation of all the educational levels included in the educational stage (1st, 2nd, 3rd and 4th), as well as a representation of both sexes in similar percentages. On the other hand, as a selection criterion, the indications provided by the educational center’s management were taken into account.

### 3.3. Variables and Instruments

#### 3.3.1. Prosocial Behavior

In turn, taking the questionnaire used as a reference, this variable is composed of the following factors: empathy, respect, social relations and leadership. The Prosocial Behavior Questionnaire (PCQ) was used to measure this variable. This is an instrument for the evaluation of PSB that is composed of four factors: empathy, respect, social relations and leadership. It consists of 54 items with four response alternatives (never, sometimes, many times and always), referring to the frequency of the described behavior. Regarding the reliability of the questionnaire, the internal consistency coefficient obtained for the entire scale was α = 0.92 [32].

Regarding data analysis strategies, after informing the center and requesting the consent of the families, and after selecting the groups of students who were going to participate in the survey, a brief explanation of the instruments to be completed was provided. Throughout the two weeks in which the information collection process was carried out, the anonymity of the students was maintained at all times.

#### 3.3.2. Executive Functions

EFECO Scale (self-report version) was used to measure this variable. This questionnaire assesses EF via an ecological approach since the report is based on the participant’s daily life behavior. The dimensions that make up the construct are flexibility, organization of materials, monitoring, inhibitory control, emotional control, working memory, initiative and planning. It is composed of 67 items that are assessed on a Likert-type scale and are rated as follows: 1 = never, 2 = sometimes, 3 = often and 4 = very often. The psychometric properties were analyzed for the Spanish version [33], finding for the total scale that its Cronbach’s alpha coefficient is α = 0.95.

#### 3.3.3. Emotional Intelligence

Bar-On’s Emotional Quotient Inventory for Youth (EQ-i:YV) [32] was used to measure emotional intelligence. According to this instrument, emotional intelligence is composed of the following factors: intrapersonal, interpersonal, stress management, state of mind and adaptation. It is a four-choice Likert-type scale questionnaire (very rarely, rarely, often, very often). The objective is the assessment of EI based on the Bar-On model mentioned in the theoretical foundation. The construct is made up of the following dimensions: intrapersonal, interpersonal, adaptability, stress management and mood. The Spanish version has been validated [34], reporting a reliability of 0.88 for the total scale.

### 3.4. Procedure

After informing the center and requesting the consent of the families, and after selecting the groups of students who were going to participate in the survey, the participants completed the measures in their group class. Each group was invited into a computer room, where one of the researchers explained to the students the main objectives of the study, reminded them that the task was completely voluntary and anonymous and thanked them for their participation. The researcher then provided them with a link to the platform where the instruments were hosted. Each student first provided their gender and age and then completed the instruments in the order presented in the previous section. The responses were stored in the program in an Excel spreadsheet, and the data file was subsequently opened with SPSS software (v25), IBM, New York, NY, USA.

## 4. Results

With the aim of analyzing the individual scores, a recoding of the scores of the items that were stated in the opposite direction was carried out. This was the case for the entire EFECO questionnaire for the assessment of EF and for some of the items of the Prosocial Behavior Questionnaire (Ccp), specifically, items 1, 12, 43 and 54 of the respect dimension and items 9 and 27 of the social relations dimension. 

Likewise, the sex variable was recoded in order to operationalize it, providing a value of 1 for male students and a value of 2 for female students.

Next, we describe the position of the sample regarding the different variables that were part of the study, that is, the sociodemographic variables of sex and age, PSB and those neuropsychological variables of EF and IE that were part of the predictive study.

The frequency corresponding to the sex variable was 75 girls (46.6%) and 86 boys (53.4%). The total sample size was 161 students.

In addition, a normality test was performed for continuous variables. Specifically, the skewness and kurtosis parameters of each variable were analyzed. As reported by IBM SPSS Statistics [35], the standard error of skewness indicates the ratio of skewness to its standard error and can be used as a test of normality (i.e., normality can be rejected if the ratio is less than −2 or greater than +2). On the other hand, the kurtosis standard error, which indicates the ratio of kurtosis to its standard error, can also be used as a test of normality (i.e., normality can be rejected if the ratio is less than −2 or greater than +2). Table 1 shows how seven of the eight continuous variables meet both of the two requirements highlighted previously. Only EF exceeded the commonly accepted thresholds, although it did so in a limited way, and thus the variable was incorporated into the parametric analyses of a robust nature.

### 4.1. Descriptive and Correlational Results

The descriptive analysis of the sample for the rest of the variables shows no apparent differences between them in the mean score, as can be seen in Table 2. The three variables obtained a moderate score. On a score of 1 to 4, EI was the variable that obtained the lowest score (M = 2.59), followed by PSB (M = 2.84) and EF (2.97). With respect to standard deviation, the results do not report a high magnitude, there being, on the other hand, apparent differences between variables in terms of the concentration of data around the mean.

The descriptive study of the dimensions of the PSB variable reported a moderate mean score with values ranging from M = 2.71 for the leadership dimension to M = 2.92 for the respect dimension. In terms of the standard deviation, again, the results do not report a high magnitude.

Regarding the dimensions of the variable EF, the descriptive analysis reported a moderate mean score somewhat higher than in the previous case with values ranging between M = 2.7 for the cognitive flexibility dimension and M = 3.17 for the monitoring dimension. Regarding the standard deviation, it should be noted that, in this case, the data reflect a greater dispersion than in the previously described cases.

After the descriptive analysis phase, we proceeded to carry out the correlational study, analyzing the bivariate correlation between the various variables of the study, calculating Pearson’s correlation coefficient and measuring the degree of covariation between the research variables in the case that they are linearly related. First, the relationship between the sociodemographic variables (sex and age) and the PSB is explored. Secondly, the correlation between EI and PSB is analyzed, and, finally, the same is done with the EFs.

As a first result to be highlighted, the variable of sex did not correlate with PSB, neither did it correlate with general PSB nor with any of the dimensions that comprise it. The same was observed with the other sociodemographic variable age. Therefore, as far as sex and age are concerned, the correlational hypothesis cannot be confirmed. 

The following Table 2 shows the descriptive results and correlations of the study, including the mean, standard deviation and Pearson correlations between the research variables.

Regarding EI, the results show a significant and positive correlation between this neuropsychological variable and PSB, both with the global variable and with each and every one of its dimensions, i.e., empathy, respect, social relations and leadership. 

Regarding the correlational studies between SF and PSB, the results obtained show a correlation between both, both with the general criterion variable and with one of the dimensions that comprise it. Specifically, it is confirmed with the respect dimension. In the latter case, the effect size (R2) is higher than that of the association between EF and overall PSB. Specifically, the common variance is 8.41% and 2.66%, respectively. 

To sum up, the study reported significant correlations between EI and PSB (global) and between EI and the four dimensions of PSB. More moderate correlations were also found between EF and PSB and between EF and the respect dimension of PSB.

### 4.2. Predictive Results

This section highlights the data obtained after the development of five predictive models, one for each factor comprising PSB, to which the one in which general PSB was taken into consideration must be added. Specifically, a linear regression analysis was carried out by the input method, including as predictors the variables described below in each of the five models:

(1) General PSB: sex, age, EI and EF (both general and its eight dimensions, i.e., monitoring, inhibition, emotional behavior, cognitive flexibility, planning, organization of materials, initiative and working memory).
(2) PSB empathy: sex, age, EI and EF (both general and its eight dimensions mentioned above).
(3) PSB respect: gender, age, EI and EF (both general and its eight dimensions mentioned above).
(4) PSB social relationships: sex, age, EI and EF (both general and its eight dimensions mentioned above).
(5) PSB leadership: gender, age, EI and EF (both general and its eight dimensions mentioned above).

In relation to the prediction of general PSB, after the introduction of the variables already mentioned, the model was configured by two predictors: EI, ß = 0.546, t = 6.800, *p* < 0.001; and cognitive flexibility (CF), ß = 0.179, t = 2.111, *p* = 0.036. The rest of the variables were excluded. The regression model obtained explains 33.3% of the variance of the overall PSB score, as reported by the coefficient of determination, R2 = 0.333. Consequently, its explanatory capacity is limited.

Regarding the prediction of the empathy dimension of the PSB, the resulting model was configured by two predictors: IE, ß = 0.468, t = 5.554, *p* < 0.001; organization of materials (FE), ß = −0.235, t = −2.097, *p* = 0.038. The remaining variables were excluded. The regression model obtained explains 26.6% of the variance of the empathy score (PSB), as reported by the coefficient of determination, R2 = 0.266. Therefore, as with the previous model, the proportion of variance in the results that can be explained by the model is limited.

Regarding the prediction of respect (PSB), the model was configured by three predictors: EI, ß = 0.495, t = 6.373, *p* < 0.001; inhibition (EF), ß = 0.422, t = 3.530, *p* = 0.001; and cognitive flexibility (CF), ß = 0.174, t = 2.128, *p* = 0.035. The remaining variables were excluded. The regression model obtained explains 38.3% of the variance of the distribution of respect scores (PSB), as reported by the coefficient of determination, R2 = 0.383. Consequently, its explanatory power is limited.

The resulting model, when considering social relationships (PSB) as a criterion variable, was configured by two predictors: EI, ß = 0.494, t = 5.784, *p* < 0.001; inhibition (EF), ß = −0.343, t = −2.606, *p* = 0.010. The remaining variables were excluded. The regression model obtained explains 25.2% of the variance of the social relations score (PSB), as reported by the coefficient of determination, R2 = 0.252. Consequently, its explanatory capacity is limited.

Finally, the prediction of leadership (PSB) offered a model configured by three predictors: sex, ß = 0.205, t = 2.564, *p* = 0.011; EI, ß = 0.394, t = 4.545, *p* < 0.001; and inhibition (EF), ß = 0.327, t = 2.448, *p* = 0.016. The remaining variables were excluded. The regression model obtained explains 23.0% of the variance of the leadership score, as reported by the coefficient of determination, R2 = 0.230. Consequently, its explanatory capacity is limited.

Table 3 shows a summary of the five regression models. In addition, information on the general statistics of the model is included in the first row. As can be inferred from the results obtained in the regression analysis, no model achieved a high explanatory capacity according to the R2 data obtained. However, it can be seen that there is one model that, with three predictors, explains almost 40% of the total variance and three other models that, with three predictors each, explain more than a third of the total variance. It is noteworthy that all five models identified significant predictors. On the other hand, EI turned out to be the common predictor in all the models and also the most powerful of all those analyzed. On the other hand, the general construct of EF was not found to be a predictor of any criterion variable; however, taking into consideration the eight dimensions that make up the construct, three dimensions of EF were found to be present in some of the models, most notably inhibition, which predicts three dimensions of PSB with moderate regression coefficients. In relation to the sociodemographic variables, only sex was found to be a predictor of the PSB dimension of leadership. Therefore, the predictive hypothesis is partially verified, taking into account the aforementioned particularities.

To sum up, the predictive analysis resulted in five regression models. It is worth noting that IE is present in all five models, being, therefore, the major predictor of the investigation. Other variables with predictive capacity turned out to be: gender (which predicts PSB leadership) and some of the dimensions of EF such as inhibition (which predicts PSB respect, PSB social relations and PSB leadership), cognitive flexibility (which predicts PSB and PSB respect) and organization of materials (which predicts PSB empathy).

## 5. Discussion

In general, EI and EF turned out to be two variables with a high impact on PSB, particularly in the case of EI and, more moderately, in the case of EF, consistent with the previous literature review [28,36]. In the case of EI, the high predictive potential [6], not only on the overall PSB but also on all the dimensions that form the criterion variable construct, became apparent [6]. The case of EF is different. Significant association with PSB was verified in coherence with previous literature [20], although its predictive capacity was found in the dimensions that compose the construct. The most striking results and how they can be interpreted from the perspective of previous studies and working hypotheses are discussed below.

Regarding the predictive hypothesis, the finding of the organization of materials as a predictor of empathy stands out. Specifically, the valence of the regression coefficient is negative. This aspect can be supported by previous studies in which the predictive capacity of EF and empathy on academic performance have been analyzed [3]. The authors of the study concluded, on the one hand, that students with lower school stress scored higher on empathy. On the other hand, the study reported that students with higher performance in mathematics scored lower on empathy. It can be interpreted that students with better academic grades also show a higher order of school materials. This fact strengthens the result obtained of the negative valence of the regression coefficient. To this finding it should be added that the planning and order dimension is the last of the EF dimensions to reach maturity, this being fifteen years on average when its maximum development is achieved [37]. Therefore, the result obtained may be conditioned by the fact that this variable is still in the process of development. Regarding the results referring to the sociodemographic variables of age and sex, it is highlighted that previous literature reports that women score higher than men on PSB [9]. Likewise, the literature reviewed reports that older people reach higher levels of PSB as this is connected to moral development [38]. This is far from the results obtained in our study. However, there is research carried out with adolescents, in the same age group as in the present study, whose results are in line with those found here. That is, with respect to sex, girls belonging to the age group considered as early adolescence between 12 and 14 years (it is recalled that the mean age of the sample of the present research is 14.1 years) do not score on PSB higher than male students [39]. The cause may be found in the compensation that occurs in this age range between the girls and their more empathic behavior and the behavior of the boys with a greater tendency to show public prosocial behaviors aimed at obtaining the approval of others [38].

On the other hand, it should be recalled that sex turned out to be a predictor of the leadership dimension of PSB. Specifically, as already reported, the female sex predicts leadership. In this regard, it is possible to establish an evolutionary hypothesis in the sense of a greater degree of maturity at these ages in girls that leads them to assume leadership and decision-making roles. On the other hand, the inclusion of gender equality in the school curriculum may be one reason why a change is taking place in the culture of adolescence.

Another result that at first glance may seem contradictory is the fact that inhibition as a dimension of EF proved to have predictive capacity on two dimensions of PSB: social relations and leadership. In the first case, it does so with a regression coefficient of negative valence. This aspect may have its argumentation in the fact that the promotion of social relationships involves necessary processes of extraversion and therefore requires a certain disconnection in inhibitory processes. In the case of leadership prediction, an adequate selection and processing of information are required to respond adequately to the needs of the context.

In any case, this aspect, like those mentioned above, can be the subject of further research and study. Likewise, in the field of foresight and considering the limitations of this research, it is proposed to carry out studies with a larger sample to allow statistical analysis of more advanced techniques such as structural equation models that facilitate the estimation of causal relationships from the statistical data.

Some limitations regarding the use of self-report measures should be highlighted. Firstly, self-protective biases are usually present in self-reports [40,41]. The respondent does not always consciously tell the truth. From the perspective of social desirability, it has been found that participants try to create a good image of themselves instead of being sincere [40]. Secondly, in closed-response self-reports, it may be the case that the respondent has a tendency to answer positively. New methods have been proposed to correct for acquiescence, particularly useful in the educational assessment of social-emotional skills [42]. It can also happen that the individual answers skew, involuntarily, toward the extreme of the scale (severity) [43] or toward the most central values (central tendency) [29]. Both types of response styles might have modified the intra-individual variability in the constructs evaluated in our study.

Another limitation of the study, which could be taken into account in future research, is the fact that EI was analyzed without considering its dimensions. In this sense, the intrapersonal and interpersonal components may yield relevant information.

Finally, the findings of this research contribute to the understanding of neuropsychological variables involved in PSB. It can be concluded that EI turned out to be a predictor of moderate intensity, being present in all the regression models of the study. In relation to EF, the study yielded information on the mild-intensity-predictor role of three of its eight dimensions. In the case of inhibition, it appears as a predictor of behaviors related to respect, social relations and leadership. In the case of cognitive flexibility, which is associated with the brain’s capacity to adapt to novel situations or contexts, it was found to have a certain predictive capacity for behaviors related to respect and, in addition, to general PSB. In relation to the organization of materials, it also demonstrated its presence in the model where empathic behaviors are included—aspects that must be taken into account in view of the specificity of the adolescent stage, such as that already mentioned regarding the sex variable, according to the results presented.

In terms of prospective or future research direction, one could carry out an enlargement of the sample, a longitudinal study, more complex statistical studies such as the use of structural equations, study of the dimensions that make up EF and extension of the study to new variables.

As for the implications of the study, it is expected to advance the improvement of the PSB of adolescents, as this factor is one of the most relevant focuses of educational research, in the case of the present study, through the acquisition of knowledge of how some neuropsychological variables, specifically IE and EF, influence behavior and attitude. At this point, it is time to transfer the research to educational praxis so that elements can be introduced in the academic curriculum to improve PSB.

These results can have direct applications in teaching planning through guidelines that incorporate the predictors that were identified with the aim of contributing to the construction of a culture of peace and the development of democratic societies. In short, neuroscience applied to education is a field in which much remains to be explored [44,45]. 

## 6. Conclusions 

The following conclusions are derived from the results obtained in this study:

1. Several variables showed significant correlation with PSB: EI and EF. On the other hand, EI also showed significant association with the rest of the dimensions that form the PSB construct: empathy, respect, social relations and leadership. In the case of EF, it also correlated with respect. Thus, the correlational hypothesis was fundamentally confirmed in relation to these pairs of variables.
2. However, the correlational hypothesis was not verified in relation to the association of the sociodemographic variables, since PSB did not correlate with sex or age.
3. EI turned out to be the predictor with the greatest predictive capacity, being present in all five regression models of the study. Thus, the predictive hypothesis would be confirmed in relation to EI.
4. In the case of EF, predictive ability was found only in the dimensions that make up the construct. This predictive analysis reported that three of the eight dimensions that make up the construct showed their predictive capacity in some of the five models: cognitive flexibility as a predictor of general PSB and of the respect dimension; organization of materials as a predictor of empathy (with negative valence of the regression coefficient); and inhibition as a predictor of respect, social relations (with negative valence) and leadership. Therefore, the predictive hypothesis was partially verified as far as EF is concerned.
5. As for the sociodemographic variables, only sex showed predictive capacity on leadership by means of a positive-valence regression coefficient on the leadership dimension of PSB. The positive valence indicates that it is the female sex that predicts leadership.

## Figures and Tables

**Table 1:** Skewness and kurtosis values.

	Skewness	Std. Error of Skewness	Kurtosis	Std. Error of Kurtosis
Age	−0.243	0.192	−0.795	0.381
EI	−0.310	0.191	−0.182	0.380
EF	−1.060	0.192	2.080	0.381
PSB	0.137	0.191	0.221	0.380
PSB empathy	0.177	0.191	−0.141	0.380
PSB respect	−0.057	0.191	−0.055	0.380
PSB social relations	−0.285	0.191	0.428	0.380
PSB leadership	0.288	0.192	−0.055	0.381

**Table 2:** Mean, standard deviation and Pearson correlations between study variables (N = 161).

Variable	*M*	*SD*	5	6	7	8	9
Sex			−0.071	−0.112	−0.102	−0.116	0.108
Age	14.1	1.18	0.152	0.118	0.130	0.103	0.122
EI	2.59	0.22	0.497 ***	0.453 ***	0.502 ***	0.420 ***	0.294 ***
EF	2.97	0.48	0.163 **	0.139	0.290 ***	−0.003	0.085
PSB	2.84	0.31					
PSB empathy	2.82	0.39					
PSB respect	2.92	0.28					
PSB social relations	2.76	0.36					
PSB leadership	2.71	0.42					

The coefficients correlating sex and prosocial behavior are of the point-biserial type. The rest are Pearson’s coefficients ** *p* < 0.01; *** *p* < 0.001.

**Table 3:** Standardized regression coefficients corresponding to the significant predictors of the five criterion variables.

Model Summary	Criteria Variables				
	PSB (General)	Empathy	Respect	Social Relations	Leadership
R (R^2^), SE	0.577 (0.333)	0.516 (0.266)	0.619 (0.383)	0.502 (0.252)	0.480 (0.230)
Predictors	0.270	0.345	0.231	0.329	0.388
sex					0.205 *
age					
IE	0.546 **	0.468 **	0.495 **	0.494 **	0.394 **
FE	-				
monitoring					
inhibition			0.422 *	−0.343 *	0.327 *
emotional behavior					
cognitive flexibility	0.179 *		0.174 *		
planning					
material organization		−0.235 *			
initiative					
working memory					

* *p* < 0.05 (bilateral); ** *p* < 0.01 (bilateral).

## Data Availability

The datasets used during the current study are available from the corresponding author on reasonable request.
